# Reverse Total Shoulder Arthroplasty for Younger Patients: A Comparable Analysis of Patients Older and Younger Than 65 Years

**DOI:** 10.5435/JAAOSGlobal-D-22-00264

**Published:** 2023-06-20

**Authors:** Marisa Deliso, Suriya Baskar, Pasquale Gencarelli, Alex Tang, Jaclyn M. Jankowski, Frank A. Liporace, Richard S. Yoon

**Affiliations:** From the Division of Orthopaedic Trauma & Adult Reconstruction, Department of Orthopaedic Surgery, Cooperman Barnabas Medical Center/Jersey City Medical Center - RWJBarnabas Health Jersey City, NJ

## Abstract

Reverse total shoulder arthroplasty (rTSA) has become a popular option for the surgical management of rotator cuff arthropathy and complex fractures of the proximal humerus. However, there is a paucity of studies evaluating outcomes, especially between patients of different age groups. The purpose of this study was to compare functional outcomes and survivorship between patients older than 65 years (o65) and those 65 years and younger (y65).

**Methods:**

A retrospective review was conducted at a single academic medical center identifying a consecutive cohort of patients undergoing rTSA between 2018 and 2020. The minimum follow-up time was 2 years. Patients were stratified into two groups for comparative analyses (y65 and o65). Patient demographics, perioperative and postoperative data, and functional outcomes were collected. A Kaplan-Meier survival analysis was conducted to determine survivorship, defined as revision surgery or implant failure.

**Results:**

Forty-eight patients were included for final analysis. Nineteen patients comprised the y65 group while 29 patients comprised the o65 group. No difference was observed in Quick Disabilities of the Arm, Shoulder, and Hand scores at baseline nor at the latest follow-up between the two groups. Patients in the y65 group had significantly greater internal and external rotation (IR/ER) from 3 months to 2 years compared with patients in the o65 group (*P* ≤ 0.05). Finally, there were no differences in revision surgery rates between the y65 group and the o65 group (11% vs. 14%, *P* = 1.0). A KM survival analysis revealed no difference in implant failure, necessitating revision surgery between the two groups at the latest follow-up (*P* = 0.69).

**Discussion:**

Despite a notable difference in the number of baseline comorbidities, there were no notable differences in functional outcomes, survivorship, and revision surgery rates between each cohort. Although both groups had a similar function initially, by 3 months postoperatively, the y65 group had markedly greater range of motion in IR and ER. Longer term survivorship is needed; however, rTSA may offer a reliable option for shoulder reconstruction even in the y65 patient group.

The reverse total shoulder arthroplasty (rTSA) procedure was originally introduced as an alternative to the standard anatomic TSA, which was lacking the desired amount of biomechanical stability.^1,2^ The rTSA was conceptualized in the 1970s when surgeons were not able to reproduce the stability granted by the rotator cuff.^2^ As more data were collected on outcomes and as technology advanced, indications for rTSA have expanded. This procedure has become a popular surgical option to treat osteoarthritis, inflammatory arthritis, rotator cuff arthropathy, and complex proximal humerus fractures.^3^

Studies evaluating shoulder arthroplasty trends demonstrate an increasing demand for these procedures.^4,5^ The number of primary shoulder arthroplasties conducted in the United States has increased by 103.7% from 2011 to 2017,^4^ and it is projected that the demand will increase by another 122% by 2040.^5^ Among this group of surgeries, rTSA is the fastest growing shoulder arthroplasty procedure in the United States.^6^ The number of rTSAs done exceeded that of the TSAs done in 2014, and this number is expected to increase by 122% by 2025.^5,6^

As more rTSAs are done, it is imperative to assess outcomes in different populations. Historically, younger patients did not receive rTSAs because of their more active and demanding lifestyle, which was thought to jeopardize the longevity of these implants. Despite this, the incidence of rTSAs in individuals younger than 65 years has been increasing since 2005.^4,7^ Multiple studies have examined the outcomes of rTSAs in younger cohorts (typically younger than 65 years),^8-11^ but data comparing rTSA outcomes between different age groups of patients are limited. The purpose of this study was to determine whether functional outcomes and survivorship of rTSA implants vary between patients who are younger and patients who are older than 65 years, regardless of initial indication for surgery. We hypothesize that patients younger than 65 years will demonstrate functional outcomes and survivorship comparable with those of patients older than 65 years.

## Methods

After receiving approval from our institutional review board, we conducted a retrospective review of 48 consecutive patients undergoing rTSA at a single academic medical center between March 2018 and March 2020. Two trauma and adult reconstruction fellowship-trained orthopaedic surgeons did all the operations in this study. Initial treatment and surgical treatment were determined primarily by surgeon preference. Inclusion criteria were as follows: (1) age 18 years and older, (2) rTSA in the setting of a proximal humerus fracture or rotator cuff arthropathy, and (3) a minimum follow-up time of 2 years. Exclusion criteria included patients with previous shoulder surgeries or surgical treatment with techniques other than rTSA.

Patients were separated into two groups for comparative analysis; the younger cohort included patients 65 years and younger (y65), and the older cohort included patients older than 65 years (o65). Data collected include patient demographics, comorbidities, indication for surgery, surgical data, range of motion (ROM), revision surgery rates, and functional outcomes such as Quick Disabilities of the Arm, Shoulder, and Hand (qDASH).

### Surgical Technique

Both surgeons place the patient in a beach chair position and use a standard deltopectoral approach. In the setting of fracture, the humeral head was removed and neck cut done if necessary. In the setting of rotator cuff arthropathy, reaming of the humeral shaft was conducted and the intramedullary alignment guide was applied before humeral head resection. In all patients, a biceps tenodesis was done. The greater and lesser tuberosities were tagged with heavy nonabsorbable suture and repaired either through bone tunnels or through the implant. Fluoroscopy and digital portable radiographs were used to check for the appropriate implant position before leaving the operating room.

### Rehabilitation Protocol

The postoperative rehabilitation protocol for both groups was adjusted at the discretion of the treating surgeon. However, protocol progression generally occurred in 4 phases (0 to 2 weeks, 2 to 6 weeks, 6 to 16 weeks, and 16 to 24 weeks). During the first 2 weeks, patients were placed in a shoulder immobilizer with an abduction pillow and made non–weight-bearing. During weeks 2 to 6, patients were allowed to start active and passive ROM exercise programs. During weeks 6 to 16, patients were made weight-bearing as tolerated and started on strengthening exercise programs. Finally, at 16 to 24 weeks, patients were allowed to gradually return to full activities.

### Statistical Analysis

Descriptive data were reported as mean ± standard deviation for continuous data or count (%) for categorical data. Independent Student *t*-tests were used to compare means between continuous variables, and chi square or Fisher exact tests were used to compare counts for categorical data. A Kaplan-Meier (KM) survival analysis was used to determine survivorship for the two groups, defined as implant failure or revision surgery. A log-rank test was used to compare survival curves between the two groups. A *P*-value of ≤0.05 was considered statistically significant. An additional subgroup analysis was conducted comparing the patients of both surgeons included in this study using the same methods. All statistical analyses were conducted using SPSS version 25 (IBM Corporation).

## Results

### Cohort

48 patients who underwent primary rTSA at our institution were identified (Supplemental Table 1, http://links.lww.com/JG9/A289). 19 patients were included in the y65 cohort, and 29 patients were included in the o65 cohort. The mean ages for our cohorts were significantly different, with the y65 group at 57.4 ± 9.1 years and the o65 group at 74.4 ± 5.5 years (*P* < 0.0001). The o65 group had a significantly greater Charlson Comorbidity Index than the y65 group (*P* < 0.0001). Outside of age and Charlson Comorbidity Index, both groups had similar demographics and baseline characteristics, including BMI (*P* = 0.41), smoking status (*P* = 0.64), and reason for rTSA (0.56). The o65 group had a significantly longer follow-up time at 3.2 ± 0.7 years in comparison with the y65 group at 2.7 ± 0.5 years (*P* = 0.007).

### Outcomes

No significant difference was observed in revision surgery rates between our two groups of patients (y65: 11%, o65: 14%, *P* = 1.0) (Supplemental Table 2, http://links.lww.com/JG9/A290). No significant difference was observed in preoperative (*P* = 0.51) and postoperative (*P* = 0.11) Quick Disabilities of the Arm, Shoulder, and Hand scores or the delta between both (*P* = 0.23). Preoperatively, there was no difference in ROM measurements between the two groups. However, at the 3-month follow-up point, the y65 group had significantly greater ROM in internal rotation (38.9 ± 15.3 vs. 29.3 ± 15.1, *P* = 0.04) and external rotation (40.8 ± 17.6 vs. 24.8 ± 12.7, *P* < 0.001). This difference persisted until the 2-year follow-up [(IR: 55.8 ± 15.7 vs. 43.3 ± 17.2, *P* = 0.01), (ER: 54.5 ± 22.2 vs. 42.1 ± 18.2, *P* = 0.04)]. The only significant difference in delta ROM occurred between the 1-year and 2-year follow-ups for flexion, where the y65 group had a greater delta than the o65 group (12.4 ± 19.5 vs. 2.6 ± 8.2, *P* = 0.05).

### Survivorship

**Figure 1:**
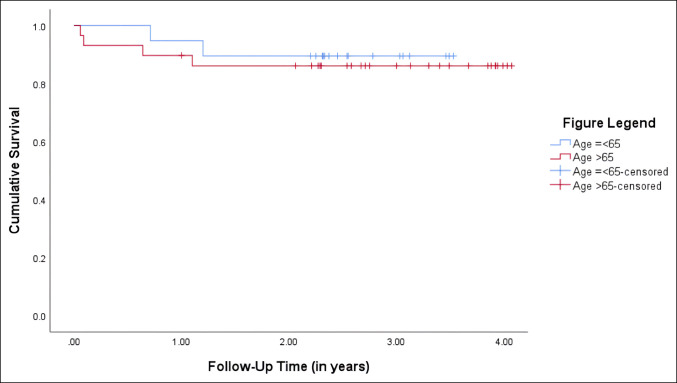
Graph showing survival curve comparison by age cohort.

Kaplan-Meier survival curves are displayed in Figure 1, which did not demonstrate any significant difference in implant survival between the two cohorts (p = 0.69) (Supplemental Table 3, http://links.lww.com/JG9/A291). The y65 group had an 89.5% survival at the 2 to 4-year follow-up period while the o65 group had an 86.1% survival rate at the 2 to 4-year follow-up.

### Subgroup Analysis

Subgroup analysis showed no significant differences between the patient cohorts of both surgeons included in the analysis other than follow-up time (3.2 ± 0.7 vs. 2.6 ± 0.4, *P* < 0.001) (Supplemental Table 4, http://links.lww.com/JG9/A292).

## Discussion

This study is among the first to compare the functional outcomes and implant survivorship after rTSA procedures in patients 65 years and younger with that of a control group consisting of patients older than 65 years. The results of this study demonstrate comparable functional outcomes and implant survivorship between both age groups of patients, validating our hypothesis.

The initial success of the rTSA was because of its ability to alleviate pain secondary to pseudoparesis in shoulders with rotator cuff tears.^1,12^ As indications expanded to include osteoarthritis, inflammatory arthritis, tumor resection, proximal humerus fractures, and infection, the number of younger patients receiving a rTSA has increased.^13^ However, the use of rTSA in younger patients has remained controversial. The primary concern was longevity of implants because studies have shown functional decline as soon as 6 years after surgery.^14^ Complication rates as high as 68% have been reported in previous studies, although there was variability in how these studies defined a complication.^15,16^ In addition, these high complication rates may be attributed to the fact that the rTSA was commonly used as a salvage procedure, and patients receiving a rTSA as a revision surgery were already a vulnerable population.^17^ As younger patients started receiving the rTSA, research revealed even higher rates of complication and revision surgery in this population. Ek et al conducted a study on 46 rTSAs done on a sample of patients with a mean age of 60 years for irreparable rotator cuff tears where they found a 37.5% complication rate and 25% revision surgery rate defined as prosthesis implant exchange, conversion to hemiarthroplasty, or resection.^9^ Wagner et al^18^ determined that every increase in age of 1 year decreases risk of revision in any shoulder arthroplasty by 3%. Similarly, Anakwenze et al found that patients older than 75 years with proximal humeral fractures had significantly lower rates of rTSA revision than patients younger than 75 years (hazard ratio = 0.45, 95% CI = 0.24 to 0.89, *P* = 0.02).^19^

Data from newer studies report more favorable outcomes for rTSA in younger patients. Matthews et al^20^ compared outcomes between a younger (younger than 65 years) and older (older than 70 years) cohort for multiple indications, where they found no notable difference in complication rates or ROM, but did find lower postoperative functional scores in the younger cohort (2 of six measurement scores). The results of our study show that starting at 3 months postoperatively, the y65 group had markedly greater ROM in IR and ER with no difference in flexion or abduction. Despite regaining ROM in two directions sooner, postoperative functional scores were not markedly different between the two groups, suggesting that the younger population had a worse perception of their functional outcome, possibly because of their more physically demanding lifestyle. Bedeir et al conducted a systematic review on patients younger than 60 years with rTSA for multiple indications and determined mean postoperative flexion and abduction to be 124.4° and 115°, respectively.^21^ In comparison, our y65 group seemed to recover better with a mean postoperative flexion and abduction of 131.6° and 122.9°, respectively, at the 2-year follow-up. A systematic review of patients younger than 65 years undergoing rTSA for multiple indications found that at 4 years, the revision surgery rate was 10%.^22^ This is consistent with the revision surgery rate of our y65 group, which was 11%. In another systematic review, Goldenberg et al found complication, revision surgery, and revision rates in patients younger than 65 years to be similar to those of older patients, concluding that rTSA is a safe and effective procedure for younger patients.^23^ Similarly, our study found a 14% rate of revision surgery in the o65 group, which was not markedly different than that of the y65 group.

Implant survival is an important consideration when interpreting the reported high rates of complication, in conjunction with the more demanding lifestyle and longer life expectancy of younger patients. Functional decline of these implants has been reported to occur 6 to 8 years postoperatively.^14,24^ Ernstbrunner et al^25^ had an average follow-up of 11.7 years in a group of patients younger than 60 years and found a 96% survival rate at 5 years and 91% at 10 years, with failures occurring because of deep infection and disassociation of the glenoid component. This is comparable with numbers published in older patients with 92 to 96% at 5 years and 89 to 93% at 10 years, with revisions caused by instability, infection, fracture, and glenoid failure.^23^ Our study did not find a notable difference in survivorship between the two age groups. Although this study had an average follow-up of only 2.97 years, this does demonstrate that short-term survivorship in younger patients is comparable with that of older patients.

There are several limitations of this study to note. The retrospective nature of this study predisposes our results to selection bias. In addition, this study includes a relatively small sample size. Nonetheless, we have included a consecutive cohort of patients, which we think increases our study's generalizability. Moreover, although both surgeons used the ideal surgical technique as previously described, rTSA is a technique-driven surgery. Although both surgeons have similar levels of experience and training, slight variability in the technique must be accounted for and may have confounded outcomes. However, subgroup analysis revealed minimal differences between the patient cohorts of both surgeons, including preoperative characteristics and postoperative outcomes. As previously noted with implant survival, the follow-up time of this study is a limitation because functional decline has been reported to begin 6 to 8 years postoperatively. For this reason, no conclusions were made regarding long-term implant survival. Additional research with larger cohorts and longer follow-up periods is required to determine whether rTSA delivers comparable results in patients younger than 65 years vs. those 65 years and older.

## Conclusion

The findings of this study support the use of rTSA in younger patient populations because of similar functional outcomes, rates of revision surgery, and short-term implant survival. Because complication rates have historically been reported to be higher in this age group, patients should still be appropriately counseled preoperatively to set appropriate expectations for their postoperative course.

## Supplementary Material
