# Supplementary material for: Reverse Total Shoulder Arthroplasty for Younger Patients: A Comparable Analysis of Patients Older and Younger Than 65 Years
Source: J Am Acad Orthop Surg Glob Res Rev. 2023 Jun 20;7(6):e22.00264. doi: 10.5435/JAAOSGlobal-D-22-00264 (PMC10284321; doi:10.5435/JAAOSGlobal-D-22-00264)
Supplement: Supplementary file 1 [file jagrr-7-e22.00264-s001.docx]

**Supplemental Table 1:** Patient Characteristics by Age Cohort

| **Characteristic^a^**  Mean (±SD) or n (%) | **y65** (n=19) | **o65** (n=29) | ***P*-value^b^** |
| --- | --- | --- | --- |
| **Age** | 57.4 ± 9.1 | 74.4 ± 5.5 | **<0.0001** |
| **Gender** |  |  | 0.06 |
| Female | 10 (53%) | 23 (79%) |  |
| Male | 9 (47%) | 6 (21%) |  |
| **Race** |  |  | 0.25 |
| White, Non-Hispanic | 12 (63%) | 16 (56%) |  |
| Black, Non-Hispanic | 1 (5%) | 7 (24%) |  |
| Hispanic/Other | 6 (32%) | 6 (20%) |  |
| **BMI** | 31.8 ± 7.6 | 30.0 ± 6.9 | 0.41 |
| **CCI** | 2.8 ± 2.2 | 5.1 ± 1.3 | **<0.0001** |
| **Smoking Status** | 1 (5%) | 4 (14%) | 0.64 |
| **Alcohol Use** | 3 (16%) | 6 (21%) | 1.0 |
| **Illicit Drug Use** | 1 (5%) | 4 (14%) | 1.0 |
| **Reason for rTSA** |  |  | 0.56 |
| Fracture | 7 (37%) | 14 (48%) |  |
| RCA | 12 (63%) | 15 (52%) |  |
| **Cement Use** | 4 (21%) | 8 (28%) | 0.74 |
| **Transfusion Rate** | 0 (0%) | 2 (7%) | 0.51 |
| **Length of Hospital Stay (days)** | 2.3±3.7 | 2.1±1.7 | 0.76 |
| **Discharge Disposition** |  |  | 0.45 |
| Home | 17 (89%) | 23 (79%) |  |
| Subacute Rehabilitation | 2 (11%) | 6 (21%) |  |
| **Follow up Time** | 2.7 ± 0.5 | 3.2 ± 0.7 | **0.007** |

*a. On initial visit. b.* P*-value by unpaired T-test for continuous variables and chi-squared test for categorical variables.
Abbreviations: SD is standard deviation. BMI is Body Mass Index, defined as mass in kilograms/height in meters squared. CCI is Charlson Comorbidity Index. RCA is rotator cuff arthropathy.*
