# Supplementary material for: Reverse Total Shoulder Arthroplasty for Younger Patients: A Comparable Analysis of Patients Older and Younger Than 65 Years
Source: J Am Acad Orthop Surg Glob Res Rev. 2023 Jun 20;7(6):e22.00264. doi: 10.5435/JAAOSGlobal-D-22-00264 (PMC10284321; doi:10.5435/JAAOSGlobal-D-22-00264)
Supplement: Supplementary file 2 [file jagrr-7-e22.00264-s002.docx]

**Supplemental Table 2:** Functional Outcomes by Age Cohort

| **Outcome Measure**  Median (IQR) or n (%) |  | **y65** (n=19) | **o65** (n=29) | ***P*-value^a,b^** |
| --- | --- | --- | --- | --- |
| **Reoperation Rate** |  | 2 (10.5%) | 4 (13.8%) | 1.0 |
| **qDASH** |  |  |  |  |
| Pre-operative |  | 69.0 ± 7.2 | 71.1 ± 14.7 | 0.51 |
| Post-operative |  | 19.7 ± 13.2 | 26.7 ± 15.5 | 0.11 |
| Δ |  | 49.2 ± 15.2 | 44.4 ± 12.5 | 0.23 |
| **ROM** |  |  |  |  |
| Pre-operative | Flexion | 34.5±31.6 | 35.3±26.3 | 0.92 |
|  | Internal Rotation | 22.4±16.6 | 20.7±12.9 | 0.70 |
|  | External Rotation | 27.4±25.4 | 19.8±13.1 | 0.24 |
|  | Abduction | 34.2±30.2 | 37.4±29.0 | 0.72 |
| Δ (Pre-operative to 3-month follow-up) | Flexion | 64.7±42.3 | 47.2±39.4 | 0.15 |
|  | Internal Rotation | 16.6±20.1 | 8.8±13.5 | 0.11 |
|  | External Rotation | 13.4±31.7 | 5.3±12.2 | 0.30 |
|  | Abduction | 53.4±35.7 | 41.7±39.0 | 0.30 |
| 3-month follow-up | Flexion | 99.2±45.0 | 80.2±37.3 | 0.12 |
|  | Internal Rotation | 38.9±15.3 | 29.3±15.1 | **0.04** |
|  | External Rotation | 40.8±17.6 | 24.8±12.7 | **0.001** |
|  | Abduction | 87.6±33.6 | 77.0±34.4 | 0.30 |
| Δ (3-month follow-up to 6-month follow-up) | Flexion | 17.6±29.6 | 20.3±21.3 | 0.71 |
|  | Internal Rotation | 7.1±4.5 | 6.6±8.1 | 0.79 |
|  | External Rotation | 4.2±14.4 | 6.4±8.0 | 0.51 |
|  | Abduction | 17.6±26.9 | 16.6±19.1 | 0.87 |
| 6-month follow-up | Flexion | 116.8±37.4 | 102.9±40.9 | 0.24 |
|  | Internal Rotation | 46.1±15.7 | 36.0±15.9 | **0.04** |
|  | External Rotation | 45.0±21.9 | 31.6±15.7 | **0.02** |
|  | Abduction | 105.3±32.0 | 95.7±38.5 | 0.37 |
| Δ (6-month follow-up to 1-year follow-up) | Flexion | 2.4±25.9 | 7.4±17.4 | 0.42 |
|  | Internal Rotation | 4.5±8.1 | 4.1±5.4 | 0.86 |
|  | External Rotation | 5.3±11.6 | 6.4±8.0 | 0.68 |
|  | Abduction | 5.5±29.1 | 16.6±19.1 | 0.49 |
| 1-year follow-up | Flexion | 119.2±45.6 | 110.3±34.1 | 0.45 |
|  | Internal Rotation | 50.5±16.5 | 40.2±17.0 | **0.04** |
|  | External Rotation | 50.3±23.8 | 37.9±18.6 | **0.05** |
|  | Abduction | 110.8±40.6 | 105.5±32.1 | 0.62 |
| Δ (1-year follow-up to 2-year follow-up) | Flexion | 12.4±19.5 | 2.6±8.2 | **0.05** |
|  | Internal Rotation | 5.3±8.6 | 3.1±3.6 | 0.23 |
|  | External Rotation | 4.2±5.3 | 4.2±5.3 | 0.96 |
|  | Abduction | 12.1±19.5 | 3.6±7.3 | 0.08 |
| 2-year follow-up | Flexion | 131.6±30.6 | 112.9±34.2 | 0.06 |
|  | Internal Rotation | 55.8±15.7 | 43.3±17.2 | **0.01** |
|  | External Rotation | 54.5±22.2 | 42.1±18.2 | **0.04** |
|  | Abduction | 122.9±27.0 | 109.1±31.9 | 0.13 |

*a.* P*-value by Unpaired T-test for continuous variables and chi-squared test for categorical variables. b. Boldened values are statistically significant.*

*Abbreviations: Δ is change, ROM is range of motion, IR is internal rotation, ER is external rotation, ABD is abduction*
