# Supplementary material for: Reverse Total Shoulder Arthroplasty for Younger Patients: A Comparable Analysis of Patients Older and Younger Than 65 Years
Source: J Am Acad Orthop Surg Glob Res Rev. 2023 Jun 20;7(6):e22.00264. doi: 10.5435/JAAOSGlobal-D-22-00264 (PMC10284321; doi:10.5435/JAAOSGlobal-D-22-00264)
Supplement: Supplementary file 3 [file jagrr-7-e22.00264-s003.docx]

**Supplemental Table 4:** Subgroup Analysis by Surgeon

| **Characteristic**  Mean (±SD) or n (%) | **Surgeon 1** (n=33) | **Surgeon 2** (n=15) | ***P*-value^a^** |
| --- | --- | --- | --- |
| **Age** | 66.9 ± 11.0 | 69.4 ± 11.1 | 0.47 |
| **Gender** |  |  | 0.64 |
| Female | 22 (67%) | 11 (73%) |  |
| Male | 11 (33%) | 4 (27%) |  |
| **Race** |  |  | 0.35 |
| White, Non-Hispanic | 16 (48%) | 12 (80%) |  |
| Black, Non-Hispanic | 6 (18%) | 2 (13%) |  |
| Hispanic/Other | 11 (33%) | 1 (7%) |  |
| **BMI** | 30.3 ± 7.2 | 31.8 ± 7.2 | 0.51 |
| **CCI** | 4.3 ± 2.2 | 4.0 ± 1.9 | 0.67 |
| **Smoking Status** | 3 (9%) | 2 (13%) | 0.66 |
| **Alcohol Use** | 5 (15%) | 4 (27%) | 0.34 |
| **Illicit Drug Use** | 2 (6%) | 0 (0%) | 0.33 |
| **Reason for rTSA** |  |  | 0.12 |
| Fracture | 12 (36%) | 9 (60%) |  |
| RCA | 21 (64%) | 6 (30%) |  |
| **Length of Hospital Stay (days)** | 2.1±2.8 | 2.4±2.3 | 0.69 |
| **Follow up Time (years)** | 3.2 ± 0.7 | 2.6 ± 0.4 | **<0.001** |
| **Reoperation Rate** | 4 (12%) | 2 (13%) | 0.91 |
| **qDASH** |  |  |  |
| Pre-operative | 69.3±10.8 | 72.4±15.0 | 0.41 |
| Post-operative | 23.8±15.2 | 24.2±14.6 | 0.93 |
| Δ | 45.4±12.1 | 48.1±16.9 | 0.52 |

*a.* P*-value by unpaired T-test for continuous variables and chi-squared test for categorical variables.
Abbreviations: SD is standard deviation. BMI is Body Mass Index, defined as mass in kilograms/height
in meters squared. CCI is Charlson Comorbidity Index. RCA is rotator cuff arthropathy. Δ is change.*
